# Breastfeeding practices in Masaya, Nicaragua: a facility based cross-sectional study

**DOI:** 10.1186/s13006-020-00273-0

**Published:** 2020-04-22

**Authors:** Aleisha M. Nabower, Elizabeth R. Lyden, Francisco J. Rodriguez, Shirley F. Delair

**Affiliations:** 1 Pediatrics, University of Nebraska Medical Center, 985525 Nebraska Medical Center, Omaha, NE USA; 2 Children’s Hospital and Medical Center, 8200 Dodge St, Omaha, NE USA; 3 University of Nebraska College of Public Health, 984355 Medical Center, Omaha, NE USA; 4 Pediatrics, Hospital Dr. Humberto Alvarado Vásquez, Mcdo Municipal 2 ½ c al E, Masaya, Nicaragua

**Keywords:** Breastfeeding, Nicaragua, Socioeconomic factors

## Abstract

**Background:**

The World Health Organization recommends exclusive breastfeeding for 6 months and total breastfeeding for at least 2 years. Despite this and multiple interventions promoting breastfeeding, early breastfeeding cessation remains high with little data as to the ongoing barriers contributing to early cessation.

**Methods:**

Two groups of Nicaraguan mothers in an urban hospital were approached to complete a questionnaire to determine what newborn, maternal, and socioeconomic factors contributed to early cessation of breastfeeding. Group 1 participants were mothers of newborns in the newborn units, while group 2 were mothers of children 5 years or younger in the emergency room and pediatric ward. Descriptive statistics summarized the data. Fisher’s exact test evaluated factors associated with early breastfeeding cessation.

**Results:**

In group 1, 97 participants were enrolled with 81% of mothers planning to fulfill the guideline for exclusive breastfeeding for 6 months. In group 2, there were 139 mothers of which 58% reported they had exclusively breastfed for 6 months. Only 25 and 27% of mothers in group 1 and 2 respectively planned to breastfeed or breastfed for 2 years. In group 1, mothers reported lack of knowledge regarding breastfeeding techniques and older mothers tended to plan for early cessation of exclusive breastfeeding. In group 2, mothers reported feeling uncomfortable with breastfeeding in public or had difficulty with latching. Cessation of any breastfeeding prior to 12 months was associated with being uncomfortable breastfeeding in public and knowing the WHO guidelines. In both groups, social media represented an expanding platform for receiving breastfeeding information.

**Conclusions:**

Interventions focusing on reaching younger mothers and addressing breastfeeding knowledge and techniques while leveraging the increasing influence of social media platforms may help improve compliance with breastfeeding recommendations.

## Background

The World Health Organization (WHO) and the Nicaragua Ministry of Health have recommended exclusive breastfeeding for the first 6 months of life since 1999 [1, 2]. After 6 months other foods should be introduced, while continuing to breastfeed up until 2 years of age [1, 2]. A 2006 study in Nicaragua found that exclusive breastfeeding for the first 5 months of life was associated with attaining optimal nutritional status and decreasing infant’s mortality [3]. Additionally, breastfeeding decreases the rate of acute otitis media, lower respiratory tract infections, and necrotizing enterocolitis [4].

A study in 1998 found that only 29.5% of Nicaraguan mothers with a three-month old were exclusively breastfeeding and 61.4% were partially breastfeeding according to a 24-h recall checklist [5]. A 1999 Mexican study associated lower rates of exclusive breastfeeding with either the mother or her significant other being the head of the household, versus another related family member. Other deterrents included perceived insufficient breastmilk supply, pain, belief that there are better sources of nutrition, and time [6]. In contrast, a lower socioeconomic status, living in the central region, and female sex of the child were significantly correlated with increased rates of exclusive breastfeeding in Nicaragua [5]. A 2015 survey of mothers in Central America demonstrated that initiation of breastfeeding within 1 hour of life and mothers with at least a primary education were associated with increased rates of breastfeeding [7]. These factors have largely been addressed by the Baby Friendly Hospital initiative that has sought to embed a culture within birthing hospitals where early exclusive breastfeeding is both supported and encouraged [8]. Despite this, a 2015 study in Leon, Nicaragua showed only 12.7% of mothers exclusively breastfed at 6 months. This study, however, did not go on to examine rates of breastfeeding past 6 months [9].

The aim of this study is to explore what factors contribute to early cessation of breastfeeding despite multiple initiatives to promote breastfeeding. Identifying potential factors will help design focused interventions to improve breastfeeding compliance.

## Methods

In this cross-sectional survey, two questionnaires were conducted at Hospital Dr. Humberto Alvarado Vásquez in Masaya, Nicaragua during the month of April 2017. Group 1 participants included all mothers 16 years and older of infants within the neonatal intensive care unit (NICU) or newborn nursery. The cut off age of 16 years was chosen given the high adolescent birth rate, with 26% of Nicaragua women having their first child before the age of 18 years, but only 4% having their first birth younger than 15 years of age [10]. Hospital births were thought to capture a large majority of the population as 93% of births in Masaya in 2011–2012 occurred at a healthcare facility, similar to the national average of 88% [10]. Group 2 included all mothers of children under 5 years of age either in the pediatric ward or the emergency department.

### Location practices

Masaya is an urban setting located in western Nicaragua and has a population just under 170,000 [11]. At Hospital Dr. Humberto Alvarado Vásquez newborns are admitted to either a shared mother/baby ward or to the NICU, with a nearby house for the mothers to stay. The hospital has been certified as a Baby Friendly Hospital, which includes: having a written breastfeeding policy and training all staff on implementing that policy; informing mothers about the benefits of breastfeeding and helping them to initiate breastfeeding within 30 min of birth; offering no other food or drink other than breastmilk to infants unless medically indicated; avoiding giving pacifiers to breastfeeding infants; and providing breastfeeding support groups for mothers [12]. Practically, this means mothers in both the NICU and newborn nursery receive teaching on the benefits of breastfeeding and steps/mechanics involved in breastfeeding from the nursing staff. Often feeds are observed by the nursing staff during the hospitalization of the infants, which allows for active coaching. For infants in the NICU unable to feed from the breast, mothers are taught how to express breastmilk and provide it to their infant via a cup. Infant formula is not provided unless medically indicated. Upon discharge from the hospital there are medical teams including nurses and doctors, who go to the homes of newborns to reinforce breastfeeding practices in addition to the educational materials available at the health centers.

### Questionnaires

The questionnaires were developed after review of the literature looking at common topics that have previously been associated with breastfeeding duration and were edited by an expert reviewer. Both questionnaires were translated into Spanish via professional translators through the University of Nebraska Medical Center (UNMC). Both questionnaires included demographics (age, education- highest level attained, employment status, relationship status, who was the head of the household, number of children), socioeconomic markers (toilet type in the home -indoor versus latrine, childcare services used, self-reported financial concerns, and transportation methods), use of medical prenatal services, breastfeeding in the first hour of life, exposure to breastfeeding, knowledge of medical recommendations for duration of breastfeeding, perceived reasons to breastfeed, perceived barriers to breastfeeding, partner and family support for breastfeeding, and the health status of the child (birthweight, prematurity, medical conditions that prevented breastfeeding, and parent’s perception of weight). Additionally, group 1 mothers in questionnaire 1 were asked how long they planned to breastfeed both exclusively and in total as well as potential barriers to breastfeeding that concerned them. Group 2 mothers in questionnaire 2 were asked how long they breastfed both exclusively and in total, barriers that they experienced regarding breastfeeding and their perception about their child’s nutritional status. Exclusive breastfeeding was explained to mothers as restricting their infant’s nutrition to breastmilk only.

A convenience sample of mothers of children within the hospital were approached by the lead investigator for consent for participation in the survey. Mothers were identified via hospital staff if they had a child present in one of the survey sites. The surveys were conducted in person Monday through Friday by the lead investigator who speaks Spanish. See the supplemental materials for the full English-version of the questionnaires. Data were collected electronically using an iPad via the Research Electronic Data Capture (RedCap) survey systems, a secure online survey database.

SAS version 9.4 was used for statistical analysis. Descriptive statistics were used to summarize the data. Fisher’s exact test was used to evaluate dichotomous factors that might be associated with breastfeeding compliance. The independent t-test was used to compare mother’s age with breastfeeding compliance. Variables significant at the < 0.10 level in univariate analysis were included in a multivariable logistic regression model. For the multivariable analysis, a backward selection method was used to determine the best subset of variables predictive of either stopping breastfeeding before 6 months (early stopping) or stopping before 24 months (late stopping). This method involved running a full model with all the predictors first and then sequentially removing factors that were least associated with the outcome until only variables significant at the *p* < 0.10 level were left in the final model. The multivariate model for early cessation of exclusive breastfeeding for group 1 mothers included age, family/friends recommending supplementation, medical professionals recommending supplementation, and not knowing how to breastfeed; while the equivalent model for group 2 mothers included poor latch and feeling uncomfortable breastfeeding in public. The multivariate model for cessation of all breastfeeding prior to 24 months for group 1 included insufficient breastmilk supply, infant illness, being uncomfortable in public, and knowing exclusive breastfeeding recommendations. For group 2 only mother's age was associated with cessation of any breastfeeding prior to 24 months so no multivariate analysis was conducted.

IRB approval was obtained from the University of Nebraska review board. The study was also authorized by the hospital governing body of Hospital Humberto Alvarado. All participants were verbally consented prior to participation in the study.

## Results

One hundred and two mothers aged 16 to 43 years (median 25) completed questionnaire 1 (group 1) and 140 mothers aged 17 to 46 years (median 27) completed questionnaire 2. However, 5 mothers in group 1 and one mother in group 2 did not provide complete information regarding duration of breastfeeding and thus were excluded from further analysis. Demographic information of both groups can be found in Table 1. Mean age was 25.2 years for group 1 mothers and 28.4 years for group 2 mothers. While 13.4% of mothers in group 1 were married, 38.2% of those in group 2 were married. Vaginal delivery was most common in both groups with 58 -60% of infants born that way. Roughly 25% of infants in both groups were premature. Mean birthweight was 3184 g in group 1 and 3252 g in group 2 (Table 1).

**Table 1:** Demographic characteristics of the surveyed population in Nicaragua

Characteristic	Newborn *N* = 97 (%)	Under 5 years old *N* = 139 (%)
Employment status		
Work outside the home	26 (26.8%)	51 (36.6%)
Housewife	71 (73.2%)	87 (62.5%)
In-home business	13 (13.4%)	19 (13.7%)
Relationship status		
Single	11 (11.3%)	24 (17.2%)
In a relationship	73 (75.3%)	61 (43.8%)
Married	13 (13.4%)	53 (38.2%)
Education level		
Incomplete primary school	21 (21.7%)	36 (25.9%)
Completed primary school	27 (27.8%)	39 (28.1%)
Completed secondary school	32 (33.0%)	42 (30.2%)
Completed university or beyond	17 (17.5%)	22 (15.9%)
Child’s sex		
Male	49 (50.5%)	67 (48.2%)
Female	48 (49.5%)	72 (51.8%)
Delivery route		
Vaginal	56 (57.7%)	84 (60.4%)
C- section	41 (42.3%)	55 (39.6%)
Preterm	24 (24.7%)	35 (25.2%)
Complications at delivery	21 (21.7%)	45 (32.4%)
House members		
Father of child	74 (76.0%)	115 (82.7%)
Grandparents	40 (41.6%)	57 (41.0%)
Aunts/uncles	19 (19.6%)	23 (16.5%)
Other children	55 (56.7%)	89 (64.0%)
Head of household		
Mother/father	65 (64.9%)	110 (79.2%)
Grandparent	30 (30.9%)	28 (20.1%)
Other	2 (2.1%)	0 (0%)
Toilet		
Indoor toilet	48 (49.4%)	65 (46.7%)
Latrine	49 (50.6%)	74 (53.3%)
Childcare		
Parents	63 (64.7%)	117 (84.1%)
Family	32 (32.8%)	41 (29.4%)
Daycare	3 (3.0%)	1 (0.7%)
Transportation to hospital		
Walk/bike	6 (6.3%)	11 (7.9%)
Taxi	32 (32.9%)	23 (16.5%)
Bus	45 (46.4%)	95 (68.4%)
Personal vehicle	14 (14.4%)	10 (7.2%)
Transportation to health center		
Walk/bike	45 (46.4%)	70 (50.4%)
Taxi	19 (19.6%)	30 (21.6%)
Bus	19 (19.5%)	29 (20.8%)
Personal vehicle	14 (14.4%)	9 (6.4%)
	**Mean (range)**	**Mean (range)**
Age (years)	25.2 (16–43)	28.4 (17–46)
Total school attendance (years)	9.30 (1–19)	8.88 (1–20)
Number of children	2.13 (1–8)	2.21 (1–7)
First prenatal visit (weeks gestation)	14.0 (1–41)	10.9 (1–40)
Birthweight (grams)	3184 (1588–4082)	3252 (1089–5670)

### Breastfeeding practice and knowledge

In group 1, 81% of mothers planned to exclusively breastfeed at least 6 months. Of the mothers in group 2, 57.9% reported that they breastfed exclusively for at least 6 months (Table 2). On average, group 1 mothers planned to exclusively breastfeed for 7.2 months, while group 2 mothers did exclusively breastfeed for 5.5 months. Over 80% of mothers in both groups breastfed in the first hour of life. Both groups were similar in reporting exclusively breastfeeding older siblings for a mean of 6.9 and 6.2 months respectively (Table 2).

**Table 2:** Interviewed mothers’ knowledge and adherence to World Health Organization breastfeeding guidelines

Characteristic	Newborn *n* = 97 (%)	Under 5 years old *n* = 139 (%)
**Breastfed first hour of life**	80 (82.4%)	118 (84.9%)
**Feeding manner**		
Bottle fed only	0 (0%)	7 (5%)
Bottle fed and breastfed	3 (3.2%)	52 (37.4%)
Breastfed only	94 (96.8%)	80 (57.6%)
**Discontinued breastfeeding sooner than had planned**	N/A	50 (36.1%)
**Mother’s knowledge of breastfeeding recommendations (# of correct answers)**		
Exclusive	60 (61.9%)	95 (68.3%)
Total	12 (12.4%)	19 (13.6%)
**I (mom) was breastfed**	84 (87.0%)	118 (84.9%)
Time I was breastfed (months)	23.6 (1–96)	19.2 (1–48)
**Mother’s adherence to exclusive breastfeeding guidelines**	Planned	Completed
Less than 6 months	18 (18.6%)	58 (41.7%)
6 months	52 (53.6%)	53 (38.1%)
More than 6 months	27 (27.8%)	28 (20.0%)
**Time exclusive breastfeeding (months)**		
Patient	7.18 (0–24)	5.51 (0–18)
Siblings	6.90 (0.5–24)	6.18 (2–12)
**Mother’s adherence to total breastfeeding guidelines**		
Less than 24 months	71 (73.2%)	102 (73.4%)
24 months	15 (15.5%)	22 (15.7%)
More than 24 months	7 (7.2%)	14 (10%)
**Total time breastfeeding (months)**		
Patient	14.9 (0–100)	15.2 (0–54)
Sibling	18.1 (2–60)	19.7 (6–36)

Group 1 mothers planned to breastfeed for an average of 14.9 months total, while group 2 mothers reported breastfeeding for 15.2 months total. Among mothers in group 1, 23% planned for a total breastfeeding duration of at least 2 years; 25.7% of their counterparts in group 2 reported total breastfeeding for 2 years or longer (Table 2). In group 2, 36.6% of mothers reported discontinuing breastfeeding sooner than they had originally planned (Table 2). Of the mothers in group 2 who reported discontinuing breastfeeding sooner than they had planned, 6.7% exceeded 2 years, 13.3% breastfed for 2 years, and 80% discontinued prior to 2 years. Of the mothers in group 1 with previous children, 91% of those who planned to discontinue breastfeeding prior to 2 years did not meet the guidelines for their previous children, while only 25% of those planning to breastfeed at least 2 years did not meet the guidelines with previous children (*p* < 0.0001). A similar pattern was observed for group 2 with 70% who breastfed for less than 2 years not meeting the guidelines for previous children versus 50% in those who breastfed for a minimum of 2 years (*p* = 0.28).

Sixty-two percent of group 1 mothers knew the WHO exclusive breastfeeding recommendations while 12% knew the total breastfeeding recommendations. In comparison, 68.3% in group 2 knew exclusive recommendations and 13.6% knew total recommendations. However, knowledge of the recommendations did not correlate with meeting them as no correlation was seen for exclusive breast feeding in either group 1 or 2 (*p* = 0.6, 0.5 respectively); and an inverse relationship was seen in group 1 for total breastfeeding: 40% of mothers who breastfed at least 24 months knew the recommendations, while 71% of those who breastfed less than 24 months knew the recommendations (*p* = 0.008). There was no correlation for group 2 mothers (*p* = 0.4).

### Breastfeeding motivation and barriers

**Fig. 1:**
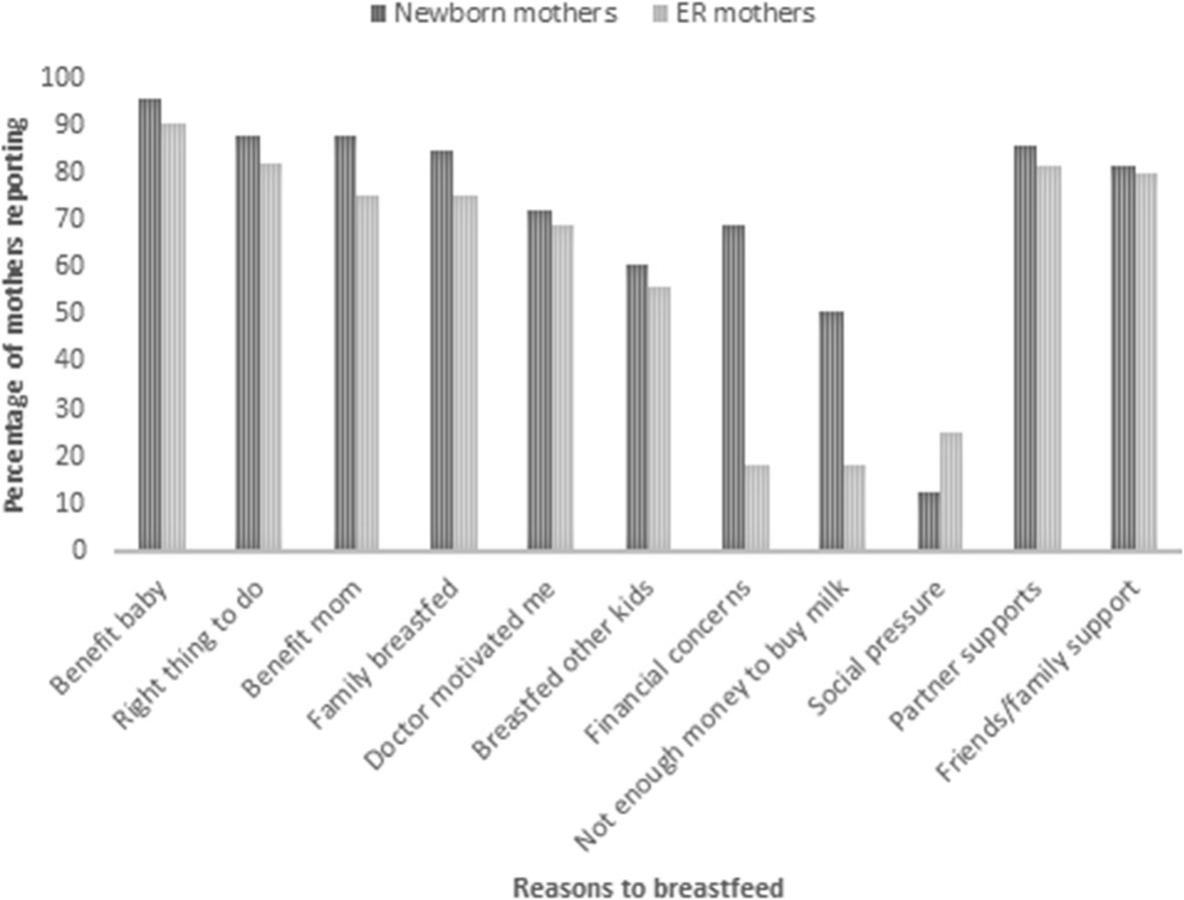
Reasons for breastfeeding reported by surveyed mothers in Nicaragua. ER- Emergency room; Family breastfed- I breastfeed because my other family members also breastfeed; Financial concerns- my family has financial concerns; Partner supports- my partner supports me breastfeeding; Friends/family support- my friends and family support me breastfeeding. *N* = 97 for newborn mothers, 139 for ER mothers

**Fig. 2:**
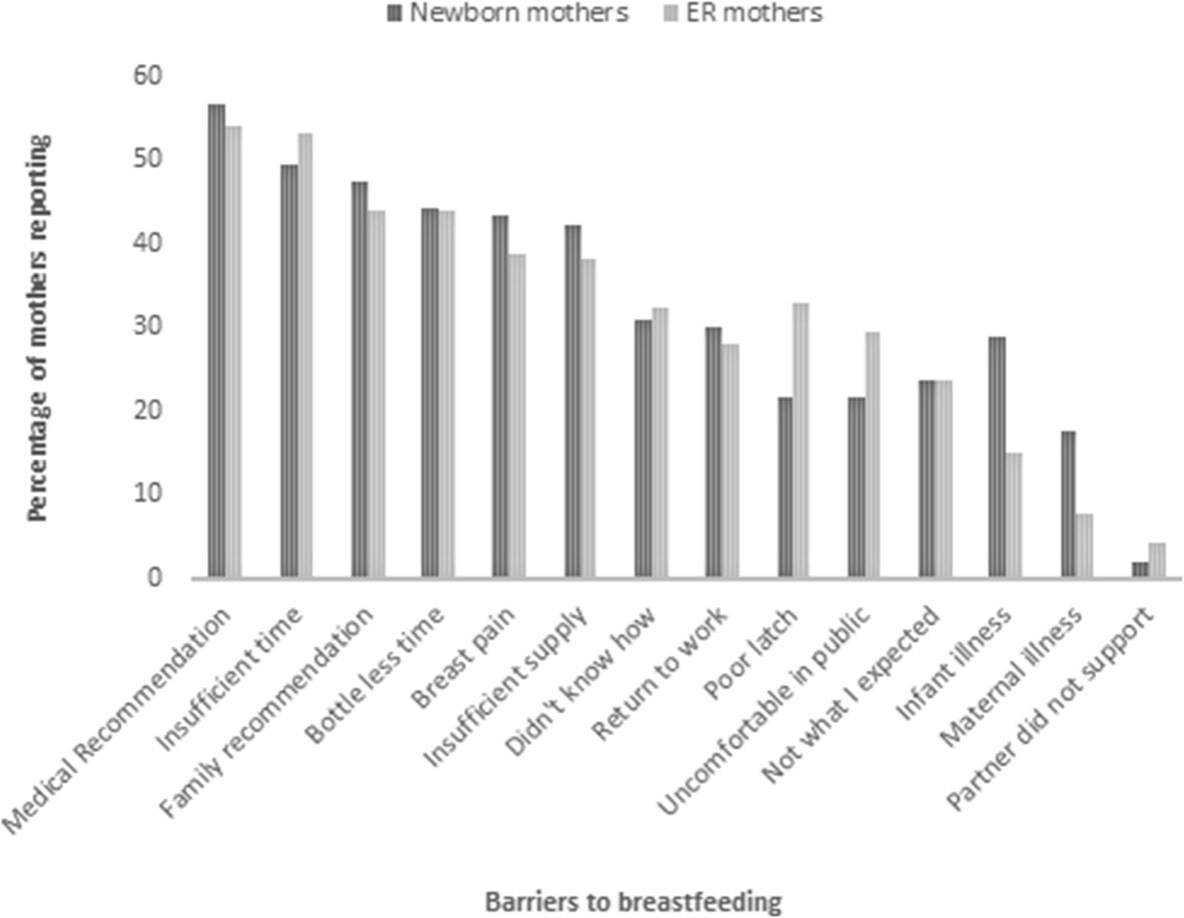
Barriers to breastfeeding reported by surveyed mothers in Nicaragua. ER- emergency room; Medical recommendation- medical personnel recommended supplementation to lactation; Family recommendation- family members recommended supplementation to lactation; Bottle less time- Giving my infant a bottle takes less time than breastfeeding, Breast pain (while breastfeeding); Didn’t know how- I did not know the proper breastfeeding technique; Return to work- I was unable to breastfeed due to having to return to work; Uncomfortable in public- I am uncomfortable breastfeeding in public; Not what I expected- breastfeeding was not what I expected; Partner did not support me breastfeeding. *N* = 97 for newborn mothers, 139 for ER mothers

Regardless of breastfeeding duration, the top five reasons mothers in both groups gave for breastfeeding were benefit to the baby, the belief that it was the right thing to do, benefits to the mother, family members breastfed, and a doctor’s recommendation to breastfeed (Fig. 1). The most common barriers were both family and healthcare personnel encouraging mothers to supplement with infant formula, insufficient time and breastmilk supply, pain, and not knowing how to breastfeed (Fig. 2).

### Predictors of early breastfeeding cessation

**Fig. 3:**
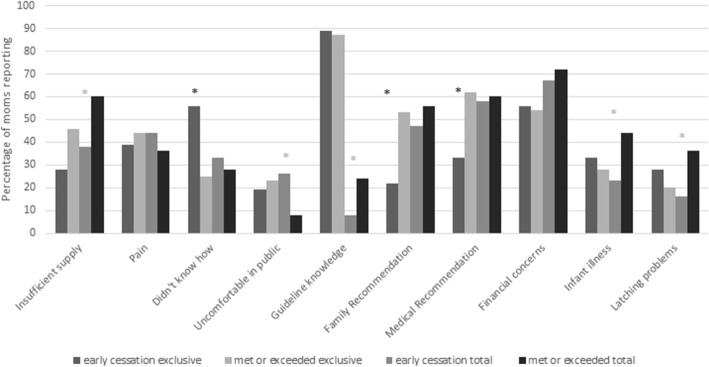
Univariate analysis of factors associated with early cessation of breastfeeding in newborn mothers. *Designates significant difference between early cessation and met or exceeded guidelines. Insufficient supply- insufficient milk supply; Pain- breast pain with breast feeding; Didn’t know how- I did not know the proper breastfeeding technique; Guideline knowledge- mother correctly reported WHO breastfeeding guidelines for exclusive and total; Family recommendation- my family recommended supplementing lactation; Medical recommendation- medical personnel recommended I supplement lactation

**Fig. 4:**
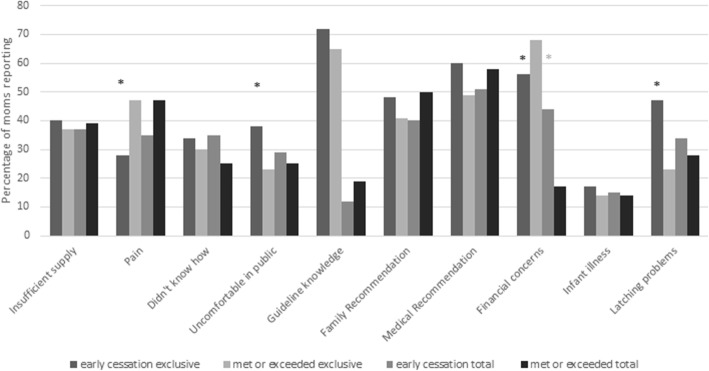
Univariate analysis of factors associated with early cessation of breastfeeding in emergency room mothers. *Designates significant difference between early cessation and met or exceeded guidelines. Insufficient supply- insufficient milk supply; Pain- breast pain with breast feeding; Didn’t know how- I did not know the proper breastfeeding technique; Guideline knowledge- mother correctly reported WHO breastfeeding guidelines for exclusive and total; Family recommendation- my family recommended supplementing lactation; Medical recommendation- medical personnel recommended I supplement lactation

Based on univariate analysis, planning for early cessation of exclusive breastfeeding in group 1 was associated with being an older mother and reporting not knowing breastfeeding techniques, (*p* = 0.02, 0.02). Family\friends or medical professionals recommending supplementation were both associated with planning to exclusively breastfeed at least 6 months (*p* = 0.02, 0.04) (Fig. 3). Group 2 mothers who reported a poor latch or being uncomfortable in public were more likely to exclusively breastfeed less than 6 months (*p* = 0.006, 0.09). Mothers reporting pain with breastfeeding or having financial concerns were more likely to exclusively breastfeed at least 6 months (*p* = 0.02, *p* = 0.02) (Fig. 4).

The multivariate model for group 1 mothers included maternal age, family/friends recommending supplementation, medical professionals recommending supplementation, and not knowing how to breastfeed. When controlling for the other variables, group 1 mothers who were older were 1.1 (95% CI 1.02, 1.22) times more likely to plan to discontinue exclusive breastfeeding prior to 6 months (*p* = 0.02), while mothers who reported not knowing breastfeeding techniques were 4.2 (95% CI 1.38, 12.93) times more likely to plan to discontinue prior to 6 months (*p* = 0.01). The multivariate model for group 2 mothers discontinuing exclusive breastfeeding prior to 6 months included poor latch and feeling uncomfortable breastfeeding in public. Group 2 mothers who reported pain were 0.3 (95% CI 0.16–0.76) times as likely to discontinue breastfeeding prior to 6 months (*p* = 0.008), while those who reported difficulty latching or feeling uncomfortable breastfeeding in public were 2.8 and 2.2 (95% CI 1.29, 5.91 and 0.99–5.02) times as likely to discontinue exclusive breastfeeding prior to 6 *months* (*p* = 0.009, 0.05).

The univariate analysis of early cessation of any breastfeeding in group 1 demonstrated that being uncomfortable breastfeeding in public or knowing exclusive breastfeeding recommendations were both associated with planning to discontinue breastfeeding prior to 24 months (*p* = 0.09, 0.008). Mothers reporting breastfeeding was beneficial to the mother, having an insufficient milk supply, difficulty latching, or having an ill infant were more likely to plan to breastfeed for at least 24 months (*p* = 0.03, 0.07, 0.05, 0.07) (Fig. 3). For group 2 mothers being an older age or having financial concerns was associated with breastfeeding at least 24 months (*p* = 0.001, 0.005) (Fig. 4).

Insufficient milk supply, infant illness, being uncomfortable breastfeeding in public, and knowing exclusive breastfeeding recommendations were included in the multivariable logistic regression model for plan to cease any breastfeeding prior to 24 months for group 1. Benefit to mother could not be included as no mothers who breastfed at least 24 months responded “no” to this survey question. Financial concerns were not included due to the proportion of mothers who did not answer the question. After controlling for the other variables, being uncomfortable breastfeeding in public and knowing exclusive recommendations were associated with being 4.6 and 4.0 (95% CI 0.95, 22.44 and 1.51, 10.66) times as likely to plan to discontinue any breastfeeding prior to 24 months (*p* = 0.06, *p* = 0.005). For group 2 mothers only, age was associated with adherence to 24 month guidelines.

### Source of information on breastfeeding

**Fig. 5:**
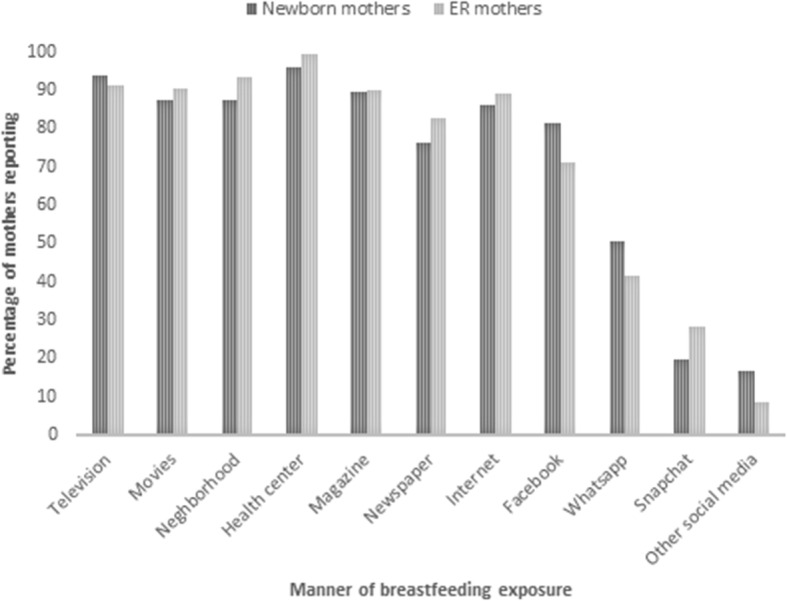
Source of breastfeeding exposure and information for surveyed mothers in Nicaragua

Mothers in both groups were exposed to breastfeeding most commonly in the healthcare centers (99 and 99.3%), their neighborhood (88 and 93.6%), and television (94 and 91.5%). Social media represents another major mode of exposure with 81 and 71.2% of mothers reporting exposure to breastfeeding on Facebook (Fig. 5).

## Discussion

Eighty-one percent of mothers of newborns in the Masaya hospital nursery and NICU reported planning to exclusively breastfeed for at least 6 months while only 58% of mothers of children less than 5 years old reported exclusively breastfeeding for 6 months. This is an increase from the 1998 results when only 30% of Nicaraguan mothers of infants 3 months and under reported exclusive breastfeeding [5] and much more than the 24.4% of mothers of infants 5 months and under reported in the United States during 2009–2012 [13]. Almost 23% percent of women in group 1 planned to breastfeed for a minimum of 2 years while 25.7% in group 2 did, both significantly more than the 7.4% of mothers in the United States [14]. Early cessation of exclusive and total breastfeeding in older siblings was associated with not meeting guidelines for the current child. This is consistent with other studies that have found past practice predicts breastfeeding duration in the younger child [15]. Not knowing how to breastfeed and poor latching were associated with early cessation of exclusive breastfeeding. This is consistent with previous studies that went on to demonstrate that interventions to change these perceptions about breastfeeding can increase duration of breastfeeding [16]. Likewise, the global Baby-Friendly Hospital initiative sought to address these issues, perhaps contributing to the increase in Nicaraguan women’s breastfeeding rates from the 1998 study [5].

The most common breastfeeding barriers reported in this study were family or friends as well as medical professionals encouraging formula supplementation and mothers having insufficient time to breastfeed. In contrast to other studies in Nicaragua and Latin America no demographic factors nor reporting insufficient time to breastfeed or insufficient breastmilk supply were found to correlate with early cessation of breastfeeding [5, 7]. Surprisingly, more mothers who exclusively breastfed reported pain than those who discontinued earlier also in contrast to previous studies [6, 17]. These mothers may have had more realistic expectations of breastfeeding to begin with, thus expecting to have some pain and therefore seing it as a normal part of breastfeeding.

Not having sufficient money to buy milk or infant formula was a concern for over half of mothers in group 1 and almost 1/3 of group 2 mothers. Practically, approximately 2/3rds and 1/3rd of women in each respective group reported planning to prolong or prolonging breastfeeding if they were having financial concerns. This is consistent with previous reports that noted a lower socioeconomic status was associated with breastfeeding longer [5]. However, no other socioeconomic markers in this study were associated with breastfeeding duration.

Mothers who breastfed a minimum of 2 years were more likely to report their baby as being born preterm. This is counter to studies in Sweden and Greece that found prematurity to be associated with early cessation of breastfeeding [15]. Perhaps there are cultural differences in the perception of these infants. It is possible that the Nicaraguan mothers perceived these infants as sicker or that they received stronger recommendations regarding continuation of breastfeeding early on that engrained the importance of breastfeeding.

Around the globe, the influence of family promoting infant formula has been reported as a common barrier to exclusive breastfeeding. Marketing of infant formula to the general public influences social norms and can decrease a mother’s confidence in the sufficiency of breast milk alone [18]. In this study, the most common mediums where women encounter breastfeeding information are health centers, within their neighborhood, and on television. Although social media does not currently have as strong an impact, it still reaches more than 70% of women. Given the impact of age on duration of total breastfeeding, this may represent a method to impact the younger mothers’ impressions of breastfeeding. While the International Code of Marketing of Breast-milk Substitutes addresses other means of formula marketing to varying degrees, the use of the internet and social media remains largely unregulated [18]. Further studies are warranted to see if the exposure that breastfeeding mothers receive through social media emphasizes negative aspects or alternative recommendations that may encourage mothers to plan to discontinue breastfeeding early. Additionally, the implementation of an intervention using social media to ensure mothers receive correct breastfeeding information, while creating another source of support for breastfeeding mothers when barriers arise, may be both wide-reaching and low-cost.

Our surveyed sample is an adequate representation of the Masaya population as evidenced by its similarities to the 2011–2012 Nicaragua National Survey, which found that 21% percent of Masaya respondents were married [10] compared to the 28% total between the two groups in the present study. Similar to our population, just over 40% of the women responding to the national survey had completed secondary education and 22% had completed university education [10]. Just over 50% of households had latrines in both studies. In the 2011–2012 national survey 11% or respondents had personal vehicles, while 10% of respondents used personal vehicles to get to the hospital in this study. On average women had 1.8 children [10] compared to an average of 2.13 and 2.21 in the respective survey groups. Mean birthweight in both groups in this study were similar to those reported by Sakisaka [3]. Thirty-three percent of deliveries reported by Sakisaka were Cesarean [10] lower than the 40–42% seen in the present study. The national survey found that 12% of mothers were exclusively breastfeeding at 5 months and that by 24–27 months 70% of infants were no longer breastfeeding at all [10].

There were several limitations to this study. First as this study was conducted in one institution over 1 month, sample size was somewhat limited, especially with regards to mothers of newborns who reported planning to breastfeed exclusively for less than 6 months. This limits the generalization of these findings both throughout Nicaragua and to other Central American countries. These results were obtained through self-reported surveys; thus, there is the potential for recall bias in Group 2. Twelve to 27% of mothers reported feeling social pressure to breastfeed and both groups of mothers may have given answers that they believed were correct according to recommendations rather than what they actually did or planned to do. Finally, as this is a cross-sectional study, while associations can be noted, we cannot determine causation.

## Conclusions

Despite multiple practice changes including the Baby Friendly Hospital initiative, the adherence rates to both exclusive and total breastfeeding guidelines remain suboptimal. Further community education is needed on both the exclusive and total breastfeeding guidelines. Additionally, continuing support of mothers who are breastfeeding is important to counter some of the barriers that mothers face. The continued use of public health centers for breastfeeding education is important, but the emergence of social media provides a potential new platform for further interventions.

## Data Availability

The datasets used and analyzed during the current study are available from the corresponding author on reasonable request.

## Abbreviation

WHO: World Health Organization
